# Next-Generation Strategies for Enamel Repair and Regeneration: Advances in Biomaterials and Translational Challenges

**DOI:** 10.1007/s13770-025-00725-w

**Published:** 2025-05-10

**Authors:** Eman M. Sedek, Ahmed A. Holiel

**Affiliations:** 1 Dental Biomaterials Department, Faculty of Dentistry, Alexandria University, Alexandria, Egypt; 2 Conservative Dentistry Department, Faculty of Dentistry, Alexandria University, Alexandria, Egypt

**Keywords:** Enamel, Tissue engineering, Remineralization, Hydroxyapatites, Biomimetic materials

## Abstract

**Background:**

Enamel regeneration and remineralization are critical for restoring enamel integrity, as natural enamel lacks the ability to regenerate due to the absence of ameloblasts. The increasing prevalence of dental caries and the irreversible nature of enamel damage highlight the need for advanced repair strategies.

**Methods:**

This review examines the latest advancements in enamel regeneration and remineralization, focusing on biomaterials, nanotechnology-based approaches, and bioengineering strategies. Google Scholar, Scopus (Elsevier), and PubMed databases were used for the selection of literature. The search included key terms such as "enamel regeneration," "biomimetic enamel repair," "stem cell-based enamel regeneration," "nanotechnology in enamel repair," "hydroxyapatite enamel remineralization," and "biomaterials for enamel remineralization."

**Results:**

Various strategies have been explored for enamel remineralization, including self-assembling peptides, dendrimers, hydrogels, and electrospun mats, each demonstrating varying success in laboratory and preclinical studies. While casein-phosphopeptide-stabilized amorphous calcium phosphate (CPP-ACP) combined with fluoride remains a widely used clinical remineralization agent, integrating CPP-ACP with nanotechnology is an emerging area requiring further research. Enamel bioengineering approaches utilizing stem/progenitor cells offer potential, though challenges remain in achieving clinical translation.

**Conclusion:**

Despite advancements, replicating the hierarchical structure and mechanical properties of natural enamel remains challenging. Nanotechnology-driven approaches, bioengineered scaffolds, and interdisciplinary collaboration hold promise for optimizing enamel regeneration techniques. Further research is necessary to enhance clinical applicability and develop scalable, effective treatments for enamel restoration.

## Introduction

Tooth enamel, the hardest tissue in the human body, is a highly organized dental tissue that covers the outer layer of the tooth crown [1]. It possesses unique mechanical and structural properties due to its high hydroxyapatite (HAp) content. In its mature form, enamel consists of 96% HAp, 1% organic components, and 3% water [2]. The enamel crystallites, typically several microns long, 50 nm wide, and 25 nm thick, vary in size depending on their depth. Enamel is subjected to various challenges, including abrasion, attrition, compressive stresses up to around 700 N [3], and, more importantly, acidic attacks from food and plaque. The outermost portion of tooth enamel is in direct contact with saliva and plaque fluid, and the surfaces of the enamel HAp crystals maintain a dynamic equilibrium with these surrounding watery phases [4].

The pH and the concentration of calcium and phosphate ions in the surrounding solution directly affect enamel dissolution. When the pH falls below 5.5, demineralization occurs as HAp dissolves, often due to acids produced by bacteria during carbohydrate metabolism or from acidic foods [5]. This process contributes to oral health problems, such as dental caries and tooth erosion, which are linked to enamel demineralization. Early detection and prevention of these issues, particularly through remineralization, are critical to maintaining enamel integrity before resorting to restorative or regenerative treatments [2]. Unlike bone, which has regenerative capabilities due to its cellular and vascular tissue, enamel has limited regenerative potential because it lacks regenerative cells (ameloblasts) and vascularity prior to tooth eruption [6].

Given the limitations of enamel’s regenerative capacity, enamel tissue engineering faces several challenges, including the complex posttranslational protein modifications required for crystal growth and the recapitulation of the unique movements of ameloblasts in organizing hydroxyapatite crystals into enamel prisms [7, 8]. Despite various attempts, no prospective clinical trial has yet explored viable cell-based enamel tissue engineering [6, 9]. As a result, enamel repair relies primarily on acellular remineralization of superficial demineralized defects [9]. Recent research has led to the development of technologies aimed at enamel repair and regeneration, offering promising solutions for restoring tooth structure. However, these strategies often lack a comprehensive understanding of the underlying mechanisms. Advancing the field requires deeper exploration of cellular, molecular, and biochemical processes, including the roles of bioactive materials, stem cell therapies, and enamel-producing cell interactions. Additionally, understanding how factors like pH, ionic composition, and mechanical properties influence remineralization and regeneration would provide valuable insights.

To facilitate advancements in enamel tissue engineering, it is essential to establish precise terminology in dental biomaterials and restorative sciences to describe the processes involved in managing dental tissue loss and damage. Terms such as regeneration, remineralization, restoration, and repair are often used interchangeably, yet each refers to a distinct process that requires clear definition. Regeneration refers to the complete restoration of lost or damaged tissue to its original structure and function, through cellular or biomaterial-based methods [9]. Remineralization, on the other hand, involves the replacement of lost minerals, such as calcium and phosphate, within demineralized enamel, effectively reversing early decay [7]. Restoration replaces lost tissue with synthetic or bioactive materials without restoring biological function of the tissue, while repair refers to mending damaged tissue to restore some degree of function but not necessarily the original structure [7]. Establishing clear definitions for these terms ensures consistency and clarity in discussing enamel regeneration strategies and their potential applications.

Advancements in adhesive technology, particularly in polymer and ceramic chemistry, have significantly improved enamel bonding strength and durability. However, the need for enamel regeneration and remineralization remains clinically relevant. Despite superior bonding performance to enamel compared to dentin, adhesive restorations are still subject to material degradation, polymerization shrinkage, secondary caries, and long-term wear [10]. Additionally, adhesive techniques often mask enamel loss rather than restore its native structure and function. Enamel regeneration presents a biological alternative that could offer superior long-term outcomes by restoring the natural properties of enamel, such as its hierarchical structure, mechanical strength, and wear resistance. This is especially relevant for conditions involving severe enamel defects, such as amelogenesis imperfecta, enamel hypoplasia, and extensive erosive wear, where conventional adhesive restorations may not provide lasting solutions [11]. Enamel regeneration, when combined with existing restorative approaches, could pave the way for more durable and biomimetic treatment strategies in the future.

This review aims to provide a comprehensive overview of the literature, highlighting novel trends and cutting-edge approaches in enamel remineralization and regeneration technologies, as well as their clinical translation. A thorough literature search was conducted up to May 2024 using multiple online databases, including Google Scholar, Scopus (Elsevier), and PubMed (United States National Library of Medicine [NLM]). The search utilized the following keywords and their combinations: "enamel regeneration," "biomimetic enamel repair," "stem cell-based enamel regeneration," "nanotechnology in enamel repair," "hydroxyapatite enamel remineralization," and "biomaterials for enamel remineralization." Retrieved articles were manually screened, and only original research and selected review articles published in English were included if they focused on enamel regeneration, biomimetic approaches, or the use of stem cells, nanotechnology, or biomaterials in enamel repair. Included studies were assessed based on their objectives, methodologies, key findings, and conclusions. The extracted data were synthesized thematically to provide a clear overview of recent advancements and emerging trends. Non-peer-reviewed sources, case reports, and studies with limited methodological clarity were excluded.

## Tooth enamel treatment modalities

**Fig. 1:**
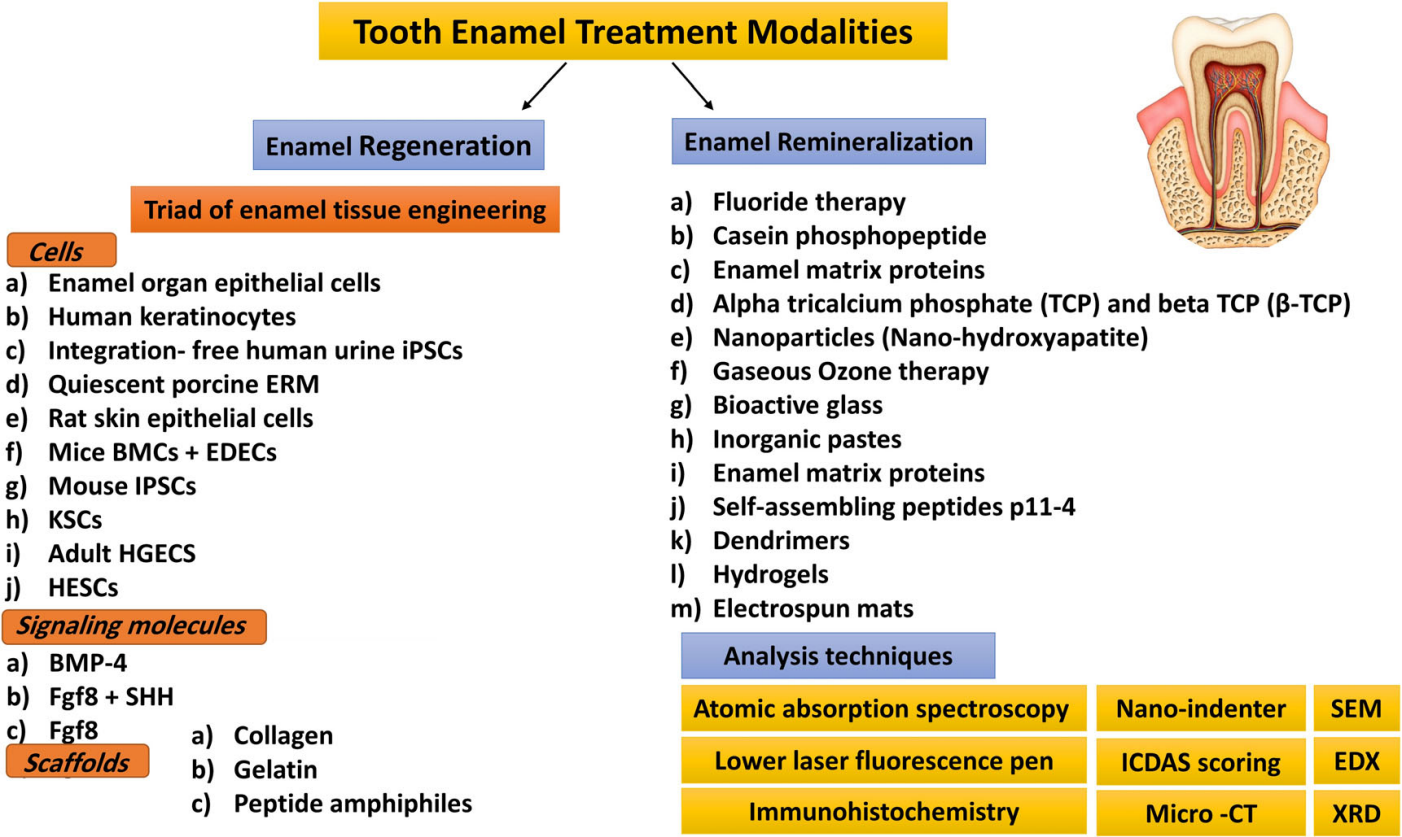
Diagram illustrates tooth enamel treatment modalities

Cellular and acellular approaches proposed for enamel regeneration and remineralization are summarized in Fig. 1. Cell-based strategies aim to mimic the natural process of enamel development given that epithelial cells can be stimulated to produce enamel postnatally during the adult life, while acellular approaches (remineralization) focus on artificially synthesizing or remineralizing enamel [7]. The most popular methods of assessment for both approaches are displayed in the Table 1.

**Table 1:** Showing the most popular methods of assessment for enamel regeneration and remineralization

Assessment techniques	
Scanning electron microscopy	
Energy-dispersive X-ray spectroscopy	Were used to evaluate the crystals formed on the demineralized enamel surface
X-ray diffraction	
A nano-indenter	Was used to evaluate the elastic modulus and nanohardness on the surface of the enamel slices
Micro- computed tomography (Micro –CT)	Was used to quantitatively measure amount of minerals after the at home application of bioactive agent
International Caries Detection and Assessment System (ICDAS scoring)	Measuring early enamel lesion changes visually
Immunohistochemistry	Using fluorescence microscope and using light microscopy
Lower laser fluorescence pen	For the early detection of tooth decay in the occlusal and proximal areas
Atomic absorption spectroscopy	Evaluate calcium and magnesium contents in tooth enamel

## Triad of enamel tissue engineering (cellular approaches)

The concept of tissue engineering relies on the employment of a triad of stem/progenitor cells, scaffolds, and growth factors to regenerate functional biological tissues. Scaffolds must be implemented with a suitable choice of cells and signaling molecules to initiate the formation of a new dental tissue that can homogenize with the surrounding tissues [12].

### Cells

Since enamel-forming cells (ameloblasts) are lost after tooth development, alternative cellular sources are required to achieve cellular-based enamel regeneration. Non dental epithelium-derived human cells, including gingival epithelial cells, induced pluripotent stem cells (iPSCs), and human keratinocyte stem cells (hKSCs) were suggested to differentiate into enamel-forming ameloblasts when combined with mouse or human embryonic dental mesenchyme [13]. This interaction provides critical signals for epithelial differentiation, as mesenchymal cells secrete enamel matrix proteins and signaling molecules. However, achieving sufficient differentiation efficiency to produce functional enamel remains a major challenge. A deeper understanding of the microenvironment, including mechanical, biochemical, and signaling cues, is essential to enhance cellular differentiation and enamel regeneration [14]. Still, only a small percentage of sub renal cultures successfully formed dental enamel. When dental epithelium and mouse embryonic dental mesenchyme were transplanted into renal capsules for 30 days, tooth-like structures with enamel and dentin formed [15]. Similarly, human keratinocyte stem cells combined with embryonic mouse dental mesenchyme, sonic hedgehog (SHH), and fibroblast growth factor 8 (Fgf8)-soaked agarose beads demonstrated ameloblastic differentiation with enamel deposition in renal capsules [16].

Hertwig’s epithelial root sheath (HERS) and epithelial rests of Malassez (ERM) cells also exhibit enamel matrix protein production [17]. HERS cells trapped in cementum express amelogenin, ameloblastin, and amelotin [18]. ERM cells cocultured with dental pulp cells differentiated into ameloblast-like cells and formed enamel-like tissue [19]. Immortalized odontogenic epithelial cells from ERM produced calcification foci and stem cell-associated gene expression when implanted in immunocompromised mice [20]. To model ameloblast behavior, immortalized cell lines offer a sustainable research tool. Viral oncogene insertion allows dental-derived cells to continuously divide while maintaining ameloblast-like characteristics [21]. These cell lines facilitate the study of amelogenesis without relying on animal-derived cells [22]. LS8, a mouse-derived cell line, models the secretory phase of amelogenesis, expressing amelogenin, ameloblastin, enamelin, and Mmp20, though it cannot form calcified nodules [23, 24]. HAT-7, a dental epithelial cell line from rat incisor apical buds, expresses both secretory (amelogenin, ameloblastin) and maturation-stage (Klk4, amelotin) ameloblast markers [25–27]. However, these rodent cell lines are not suitable for human enamel repair [7]. Despite these advances, no human clinical trials have yet utilized stem cells for enamel regeneration. A summary of different cell sources for enamel regeneration is summarized below (Table 2).

**Table 2:** Showing a summary of different cell sources and scaffold-based strategies for enamel regeneration

Cell sources	Scaffold-based strategies	Outcome	References
Third molar porcine tooth bud	Cells were seeded onto biodegradable collagen **coated PGA/ PLLA and PLGA scaffolds** and grow in rat hosts for 20 to 30 weeks	Recognizable tooth structures formed that contained a morphologically correct enamel organ containing fully formed enamel	[28]
Pig tooth bud tissues	Cell-seeded biodegradable **PGA/PLLA and PLGA scaffolds** were grown in the omenta of adult rat hosts for 12 wks	Bioengineered tooth tissues exhibited well-formed dentin, enamel, and pulp tissues	[29]
Tooth germ from porcine third molar	**Collagen coated PGA fiber meshes** with cells was implanted into rat omentum	Enamel-covered dentin	[30]
Porcine third molar teeth at the early stage of crown formation	Cells were seeded onto **collagen sponge** then implanted into the omentum of rats up to 25 weeks	Tooth production including enamel organ, dental papilla and Odontoblasts were also identified	[31]
Odontogenic epithelial cells and dental mesenchymal cells	Cells were seeded onto **collagen sponge scaffolds**, and transplanted into athymic rats for 4 weeks	Enamel-dentin comple	[32]
Ameloblast‐like cells and primary enamel organ epithelial cells	Cells were cultured within **peptide amphiphile hydrogels** then were injected into the enamel organ epithelia of mouse embryonic incisors	Scaffolds seeded with cells functioned as instructive signals for enamel formation	[33]
Dental stem cells derived from enamel and pulp organ	Cell-seeded **absorbable gelatin Dental Sponge strips** were transplanted into mandibular defects in a minipig for 12 and 20 weeks	Hybrid tooth-bone tissues containing enamel, pulp, dentin and bone, cementum and periodontal ligament	[34]
HAT-7 cells and hDPSCs	**Collagen I and chitosan blends scaffolds** seeded with cells were implanted subcutaneous in immunocompromised CD-1 mice	Enamel and dentin formation	[35]
Epithelial cells derived from human embryonic stem cells ( hESCs)	*In vitro* study using hESCs to human ameloblast-lineage cells, to determine their potential use as a cell source for enamel regeneration	hESCs a promising potential cell source for enamel regeneration	[36]
Adult human epithelial stem cells and mesenchymal stem cells	Subcutaneous cotransplantation on **PLLA scaffolds** in nude mice	Heterotopic ossicles with ultrastructure of dentin, enamel, cementum, and bone	[37]
Induced pluripotent stem (iPS) cells	*In vitro* study using medium conditioned by cultured epithelial cell rests of Malassez (ERM) cells and **gelatin-coated dishes** to stimulate iPS cells to differentiate to ameloblast -like cells	iPS cells can be induced to expand to ameloblast-like cells for later enamel regeneration	[38]
Human keratinocyte stem cells	Cells were treated with growth factors- soaked **agarose beads** into recombinant chimeric tooth germs, then were subjected to kidney capsule culture in nude mice	Tooth-like structures with intact enamel prisms	[16]
Human embryonic stem (hES) cells	hES cells to differentiate into dental epithelium, then the generated cell was mixed with embryonic day 14.5 mouse dental mesenchyme (DM) and transplanted into the renal capsules of nude mice	Teeth with enamel and dentin had formed on the kidney	[15]

### Signaling molecules

The key rationales for the involvement of signaling molecules in enamel regeneration are regulating enamel deposition [39], preserving stem cell niche [40], maintaining the equilibrium between stem cell proliferation and differentiation towards ameloblast lineage [41], activating intracellular pathways (e.g., MAPK, Notch, and β-catenin signaling) which regulate ameloblast proliferation, differentiation, and maturation [42], and acing as mesenchymal signals for ameloblast induction [43]. Numerous signalling molecules, such as fibroblast growth factor (Fgf), sonic hedgehog (SHH), wingless (Wnt), bone morphogenic protein (BMP), and transforming growth factor β (TGF-β), have been shown to be involved in the epithelial mesenchymal interactions that take place during odontogenesis [44]. Enamel deposition in mouse incisors is regulated by activin, BMP, and Fgf signals [39], while TGF-β1, BMP-4, and BMP-2 contribute to ameloblast development [43, 45]. FAK-YAP-mTOR signaling maintains the balance between stem cell proliferation and ameloblast differentiation [41]. BMP signaling is critical for enamel formation [46] and ameloblast differentiation [43]. SHH signaling sustains the molar cervical loop stem cell niche [40] and promotes ameloblastic maturation in the epithelial intermedium layer [42, 47]. Controlling these molecules may contribute to the production of progenitors of the ameloblast lineage that resemble odontogenic epithelial stem/progenitor cells and may be incorporated into enamel regeneration approaches (Table 3).

**Table 3:** Displaying a list of the various signaling molecules involved in enamel regeneration

Signaling molecules	Rationale	References
Sonic hedgehog (SHH)	Support ameloblastic differentiation and maturation in epithelial stratum intermedium cells	[42]
Fibroblast growth factor (FBF-2)	Regulates cell differentiation and matrix secretion at the bell stage	[48]
Bone morphogenetic protein (BMP-2) TGF-β1, and BMP-4	Ameloblast induction	[43]
Bone morphogenetic protein (BMP)	Crucial for ameloblast differentiation and enamel formation	[46]
Activin, BMP, and Fgf	Regulate enamel deposition	[39]
Transcription factors (Msx2 and Sp6)	Important roles in amelogenesis	[44]
BMP- SHH	Preserve stem cell niche	[40]
Globoside (Gb4)	Promote the differentiation of dental epithelial cells into ameloblasts	[49]
FAK-YAP-mTOR	Maintains the equilibrium between stem cell proliferation and differentiation towards ameloblast lineage	[41]
Runx2-Nfic-Osx	Regulates ameloblast differentiation and enamel formation	[50]
TGF-β1/ RUNX2	Enamel mineralization	[51]
Transcription factor AmeloD	Stimulates epithelial cell motility essential for tooth morphology	[52]
Serotonin	*In vitro* production of a biomimetic enamel‐like material	[53]

**Fig. 2:**
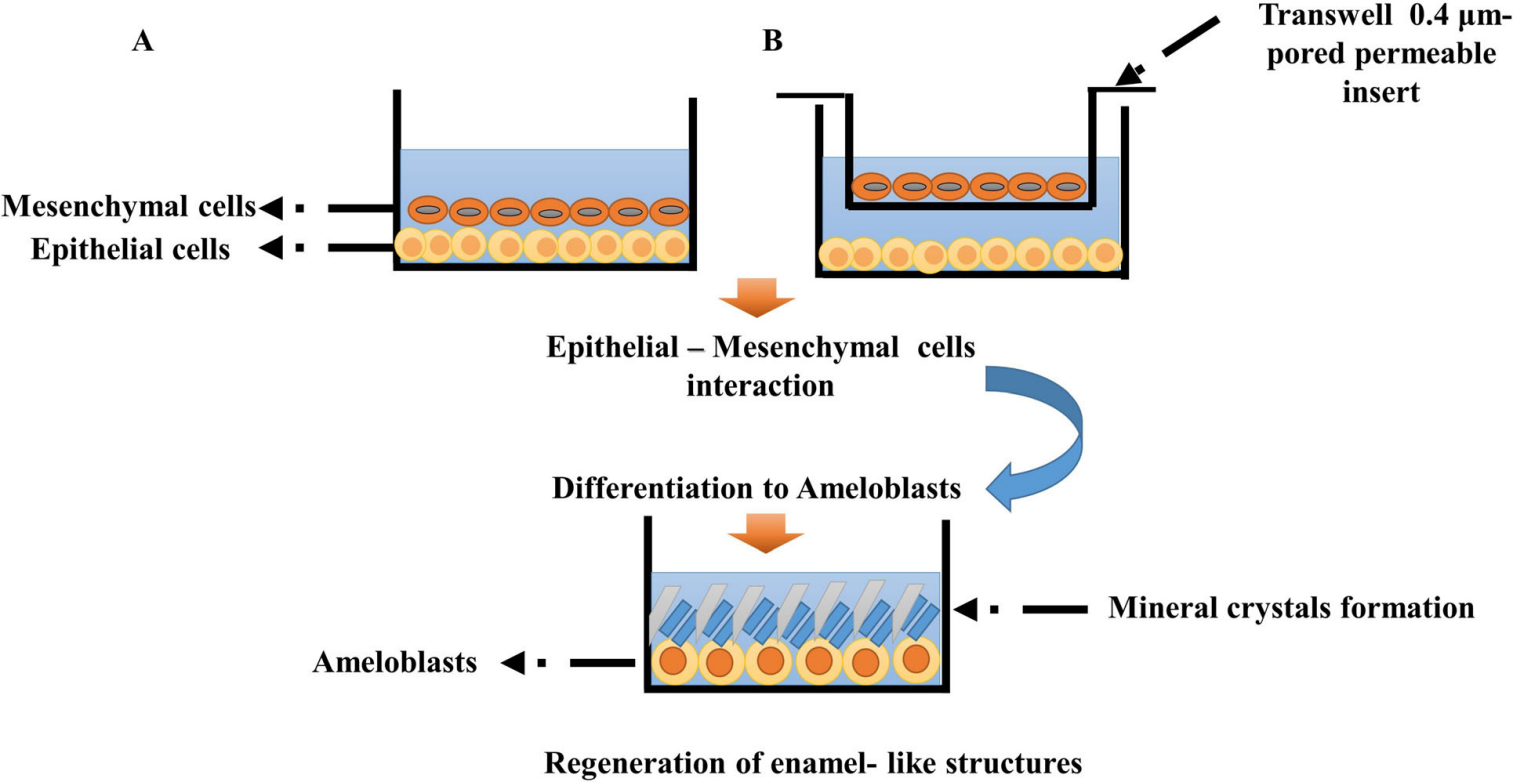
Different co-culture models: **A** 2D direct contact model, **B** 2D indirect contact model. These created models can be utilized to investigate the role that epithelial–mesenchymal signaling plays in ameloblast differentiation, as well as the formation and mineralization of enamel

Co-culture systems: Epithelial–mesenchymal interactions, which normally occur throughout tooth formation, are another crucial factor for enamel regeneration. Utilizing two- or three-dimensional (2D or 3D) co-culture systems is one way to stimulate ameloblast development and enamel production by providing such signals (Fig. 2) [32]. Transwell devices provide non-contact co-culture models wherein a single cell type is seeded into a 0.4 μm-pored permeable insert and positioned on top of wells plated with the other cell type [54]. According to this model, without actual cell-to-cell contact, soluble signaling molecules generated by each kind of cell work as inducers, influencing the function of other cells [54]. In a similar manner, mesenchymal-derived dental pulp stem cells and dental epithelial cells (HAT-7) were seeded into a three-dimensional multilayered macroscale biomimetic co-culture system applying chitosan and type I collagen to mimic epithelial mesenchymal interactions. This technique allowed mesenchymal and epithelial cells to co-culture, and the two cell types moved in different directions and Calcium crystals were observed [35]. These systems are straightforward and simple to use, however they offer a 2D monolayer of cells that are not the same as those found *in vivo* in terms of gene expression, morphology, and cell functions. Moreover, the direct cell–cell interactions are important for ameloblast differentiation [55]. The creation of a 3D co-culture microenvironment that resembles *in vivo* tissue architecture while allowing for naturally occurring cell morphogenesis is still challenging [55].

### Scaffolds

Scaffolds have been created using both natural and synthetic biomaterials in the form of hydrogels, sponges, and meshes for promoting enamel or enamel-dental tissue complex regeneration. By supplying cell–matrix signals, scaffolds aid in the creation of dental tissue [12]. Ideally, they should be able to resemble the extracellular matrix, interact with cells to promote cell attachment, proliferation, and differentiation, disintegrate over time, possess appropriate mechanical properties, and permit diffusion of waste materials and nutrients as well as cells and growth elements [56]. The most common scaffolds used for enamel regeneration are collagen, gelatin, polyglycolic acid/Poly-L-lactic acid (PGA/ PLLA), polylactide-co-glycolide (PLGA), and peptide amphiphiles. These scaffolds have been used in a variety of techniques, including solvent casting and porogen leaching to create porous scaffolds [28, 29], self-assembled nanofibers [33], multi-layered hydrogel matrix [35], nanofibers [37], fiber mesh [30], sponge strips [34], and sponge scaffolds [32].

A major challenge in enamel regeneration is replicating its natural microstructure by properly organizing hydroxyapatite crystals at the nanoscale within a scaffold [11]. Advancing scaffold design to better control the biochemical and physical properties of the regenerative environment is essential for overcoming current limitations in enamel tissue engineering [57]. The first attempt at bioengineered tooth structures involved seeding dissociated porcine third molar tooth bud cells onto collagen-coated polyglycolate/poly-L-lactate (C/PLLA) and poly(lactide-co-glycolide) (PLGA) scaffolds shaped like human teeth, which were then implanted into the omentum of athymic rats [28]. Both scaffold types supported tooth formation [29]. Although these engineered teeth contained pulp, dentin, enamel, and tissues resembling HERS and cementoblasts, they remained significantly smaller (2 × 2 mm) and did not fully match the scaffold's original shape [28]. Further approaches used branched arginine–glycine–aspartic acid–peptide amphiphile nanofibrous structures (BRGD-PA) with ameloblast-like LS8 cells and primary enamel organ epithelial cells (EOE). This system enhanced ameloblast differentiation, proliferation, matrix production, and mineralization [33]. After implantation into the renal capsule of mice for eight weeks, mineralized nodules and enamel pearls formed. BRGD-PA functioned as an artificial matrix, facilitating ameloblast differentiation through cell–matrix interactions that mimic natural dental mesenchyme signaling [58]. A summary of the scaffold-based enamel regeneration approaches is summarized below (Table 2).

Enamel regeneration is a critical objective; however, enamel tissue engineering remains a relatively novel field, facing several challenges. One is that, in contrast to native enamel, the arrangement of enamel prisms in regenerated tissue is somewhat asymmetrical [31]. Furthermore, the regenerated crowns are small and do not fit the scaffolds' size [28]. The engineering of enamel tissue is further complicated by the intricate posttranslational protein processing required for crystal formation and the unique way in which ameloblasts shape hydroxyapatite (HAp) crystals into enamel rods [9]. Moreover, the regenerated enamel-dentin complexes, which place enamel inside dentin in round or linear formations, do not replicate the morphogenetic characteristics of natural dental crowns [59]. Thus, further research is essential to understand how to regenerate enamel tissue while precisely controlling both its shape and size.

## Innovative trends and cutting-edge approaches for enamel remineralization (acellular approaches)

The process of tooth remineralization has been the subject of years of research, which has led to the creation of technologies that may either decrease enamel demineralization or increase enamel remineralization, thereby have the potential to improve oral health. There are two categories for tooth remineralization:

### Methods for effectively applying and utilizing the remineralizing agent

#### Laser aiding mineralization

**Fig. 3:**
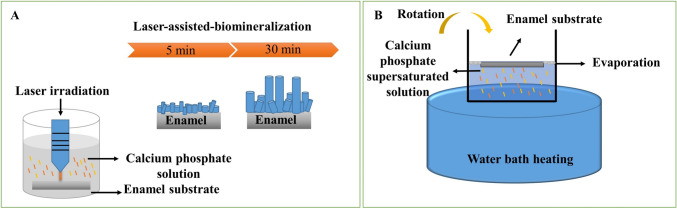
**A** Laser-assisted mineralization, **B** Rotary evaporation approach. Superficial enamel remineralization and repair can be re-established chemically by using these types of approaches, which can assist in enamel caries prevention strategies

Lasers have been widely applied in dentistry for soft tissue cutting as well as bleaching of the teeth. Because of their photothermal attributes, they can assist in crystal growth by warming the surrounding area and changing the reaction conditions into a hydrothermal oven. Thus, lasers can assist in preventing dental calculus from forming by accelerating the mineralization of dental enamel and controlling the formation of HAp crystals in the affected areas (Fig. 3A) [60]. However, the primary drawback of this method is that the overheating effect of the diode laser may damage the dentinal and pulpal layers' nerve cells. Thus, additional research is required in order to confirm whether switching the laser source is effective [61]. Femtosecond pulsed lasers (fs) are another kind of laser that can be used to remineralize enamel by sintering artificial fluorapatite powder, which is placed to the surface of demineralized enamel and contains iron oxide nanoparticles in chitosan. Like a thermal antenna, these nanoparticles were able to absorb laser photons, disperse the heat locally to fluorapatite crystals, and cause densification into a dense layer that adhered to the original enamel [60].

#### Rotary evaporation

It is a simple and effective method for regulating crystal formation and regenerating enamel-like structures on a variety of substrates, such as dentin and dental enamel. In contrast to alternative methods like hydrothermal approaches, which only provide a restricted thickness of the regenerated layer and a very slow rate of HAp crystal growth (less than 1 μm per day), rotary evaporation can produce highly ordered structures at a controlled thickness very quickly (Fig. 3B) [7]. HAp crystals are produced in the remineralizing solution containing calcium, phosphate, and fluoride minerals using silk fibroin, which was used as a modulator of crystal formation because of its resemblance to the structure of rotational evaporation. The microstructure (crystal size, shape, organization) and mechanical characteristics of the regenerated enamel-like crystals made with this technique were comparable to those of native enamel [62].

#### Electrically enhanced remineralization

**Fig. 4:**
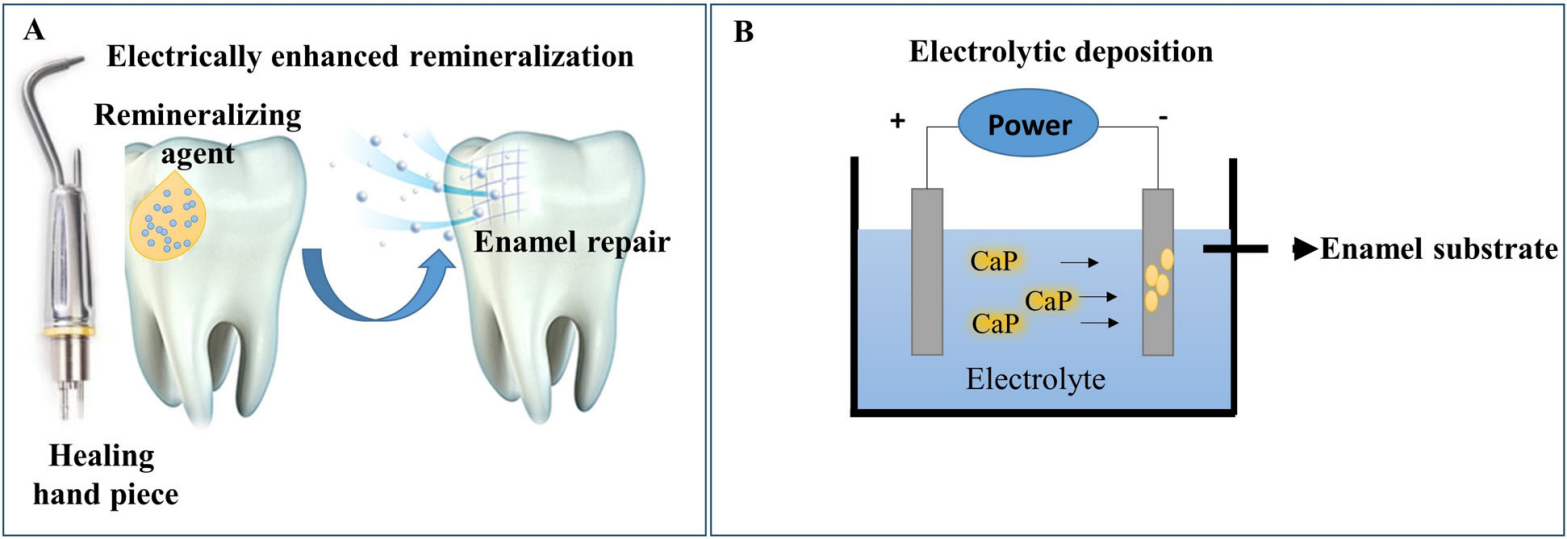
**A** The repair method using a small 'healing hand piece' applied to the tooth's decayed surface together with remineralizing agents, **B** Electrodeposition method. Superficial enamel remineralization and repair can be re-established chemically by using these types of approaches, which can assist in enamel caries prevention strategies

An innovative clinical method called electrically accelerated remineralization can quickly and effectively remineralize dental cavities without the need for drilling or fillings [63]. By using a painless procedure with no injections, drills, or restorative materials, this method can be used to heal the complete depth of initial-stage or moderate carious lesions [63]. Moreover, this technique can be used to maintain the integrity of the tooth by protecting the healthy portion of the tooth during the process. Using a small 'healing hand piece' applied to the tooth's decayed surface (Fig. 4A), the procedure consists of two steps: first, remineralizing agents (a paste or liquid) are applied to the carious lesions to act as a reservoir of minerals; second, an electric field is briefly applied to hasten the mineral agents' entry into the lesion [64]. Fluoride, CPP-ACP, tricalcium phosphate, and inorganic amorphous calcium sodium phosphosilicate have been used as remineralizing agents in this technique [7].

#### Electrodeposition

It is a simple and reasonably priced technique for depositing a uniform layer on substrate under an electrical field (Fig. 4B) [65]. The two fundamental types of electrodepositions are electrolytic deposition (ELD) and electrophoretic deposition (EPD). Compared to the EPD, the ELD creates coating layers that are thinner [65]. ELD method's capacity to simultaneously induce the precipitation of calcium phosphate (CaP) and self-assembled amelogenin proteins under physiological to enhance the enamel-like composite coatings' such as mechanical qualities and crystal development [66]. This method has the benefit of coating surfaces with CaP, or mineral crystals, at a relatively low temperature with regulated crystallinity, even if the substrates have porous or uneven features [67]. Additionally, the slow elevation of the local pH in the area of the cathode can trigger the amelogenin protein self-assembly process, CaP supersaturation, and crystal nucleation on the cathode [68]. Unfortunately, the potential for this approach to be translated into clinical applications is limited by the high electric field condition used in the procedure [69].

#### Immunization against cariogenic bacteria

Caries vaccines are another strategy for the prevention of dental caries has been established during the past 40 years. The primary targets of the caries vaccines development are Streptococcus mutans and Streptococcus sobrinus [70]. It is possible to actively or passively immunize against cariogenic microorganisms [71]. Target antigens are used in active immunization to stimulate the immune system of the host to produce endogenous antibodies [71]. Active vaccinations are longer-acting and more effective, but they might cause detrimental immune responses that could be fatal [72]. Exogenous antibody delivery is used in passive immunization, which is a safer approach but requires repeated administrations due to its less effective and transient protection [70, 71]. Furthermore, immunological responses are frequently triggered by non-human antibodies used in passive vaccinations [73]. Using recombinant DNA technology, antigen-binding fragments (Fabs) against Streptococcus mutans and Streptococcus sobrinus were synthesized to prevent caries formation in a rat model. These Fabs have the potential to be engineered for passive immunization, which would inhibit immune responses to non-human antibodies [73]. Further research is required to determine whether immunizations are beneficial in the long run for treating dental caries. Additionally, it is thought that by preventing cariogenic bacteria, a mixture of enamel matrix proteins or any remineralizing agent and antibodies may be able to treat dental caries in a carious tooth while also preventing the caries from progressing further.

### In situ remineralization, or the regeneration of a structure like enamel by imitating its functions of an organic matrix (Clinical applicable agents) (Table 4)

**Table 4:** Summary of current technology (commercially available remineralizing agent)

Material—Trade name	Category	Ref
MI Varnish (GC America, Inc, Alsip, IL) ProSeal sealant (Reliance Orthodontic Products, Itasca, IL) Enamelast (Ultradent Products Inc., South Jordan) Duraphat (Colgate, united states) Fluor Protector S (Ivoclar Vivadent's)	Fluoride varnish Can mineralize non-cavitated lesions only	[74]
Clinpro™ Tooth Crème (3 M ESPE.)	Tricalcium phosphate fluoride Have potential remineralization for white spot lesions	[75]
MI-Paste-Plus® (MPP)- Recaldent™	Casein-phosphopeptide-amorphous-calcium-fluoride-phosphate (CPP-ACFP) Have potential remineralization for early enamel lesions and white spot lesions	[74, 75]
Cerasorb® Bio-Resorb® Biovision®	Beta Tricalcium phosphate Enhance calcium content of saliva and plaque	[76]
ApaCare® & Repair (Cumdente GmbH Paul-Ehrlich-Straße, Germany)	Nano-hydroxyapatite gel Sources to release calcium/phosphate ions and increase the supersaturation of HAP in carious lesions	[77]
OzonyTron apparatus (Mymed, Germany)	Ozone therapy Remineralize caries lesions by reducing the level of caries-associated microorganisms in dental plaque	[78]
NovaMin™	Bioactive glass Form a protective hydroxyl carbonate apatite layer	[79]
Curodont™ Repair (vVARDIS, Switzerland)	Self-assembling peptides P11-4 Superior remineralization with uniform mineral deposition	[80]
Emdogain® (Straumann® Biora AB, Switzerland.)	Enamel matrix protein containing hydrogel Promote enamel prism-like formation on demineralized enamel *in vitro*	[81]

#### Fluoride therapy

**Fig. 5:**
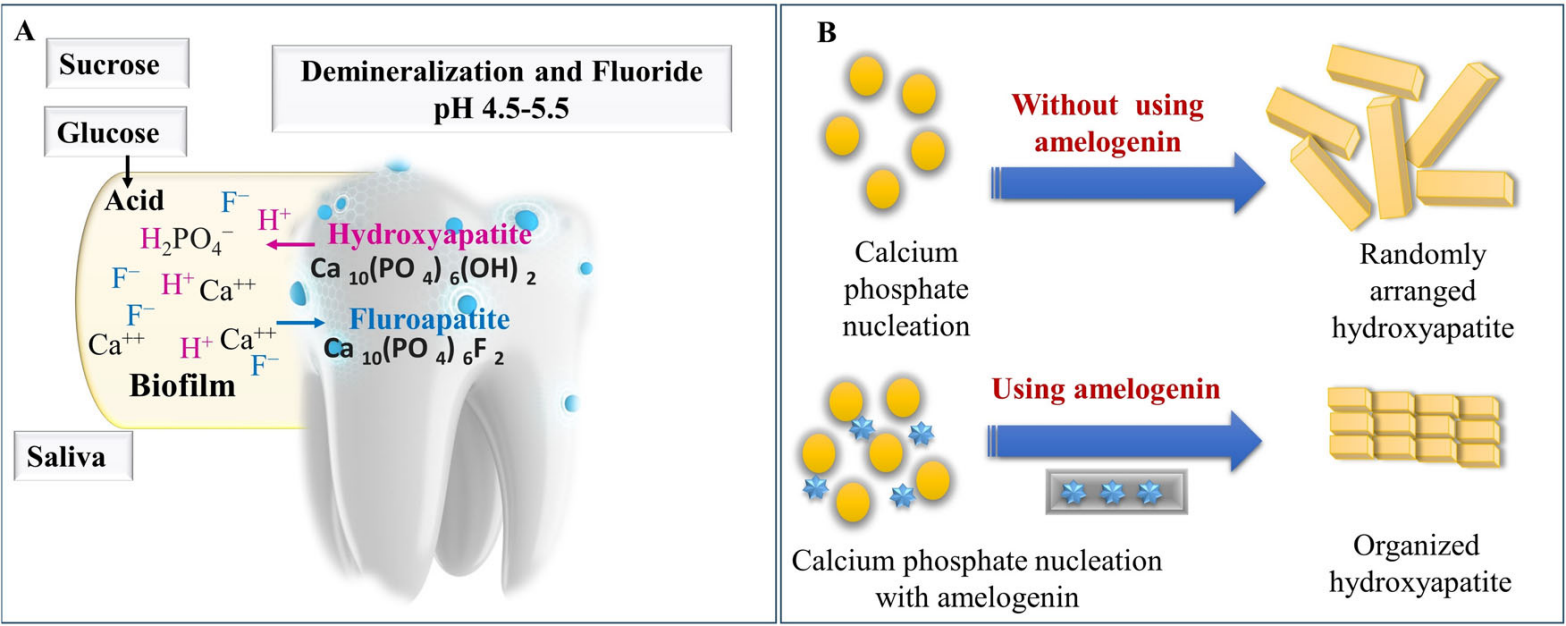
**A** The mechanism by which fluoride inhibits demineralization, **B** Amelogenin, following calcium phosphate nucleation, can facilitate the formation of organized needle-like Hap nanocrystals

Fluoride therapy is one of the strategies that has been used historically to remineralize enamel and prevent dental cavities [82]. Saliva serves as an inherent remineralization solution since fluoride is present there in extremely small amounts—roughly sub-ppm [82]. Fluoride works primarily via four topical mechanisms which include [76, 83] 1. Under cariogenic (acidic, 5.5) pH, which is generated due to the process by which carbohydrates are changed into acids via bacteria in the plaque biofilm, phosphate ions in the biofilm drop below normal, and the enamel's hydroxyapatite dissolves to restore equilibrium. In the presence of fluoride, the hydroxyl groups (OH^−^) in the crystal lattice were replaced by fluoride ions (F^−^), which led to the production of fluorapatite, which has a lower solubility and a greater resistance to acid attack compared to HAP crystals (Fig. 5). 2. By bringing calcium and phosphate ions together, fluoride accelerates the development of new fluorapatite crystals, improving remineralization. 3. By interfering with the synthesis of phosphoenol pyruvate (PEP), a crucial intermediary of the glycolytic pathway in bacteria, it suppresses the action of acid-producing carious bacteria. 4. The fluoride remains on dental hard tissue and in the dental plaque to reduce demineralization and improve remineralization. However, the high concentration of fluoride may have detrimental impacts because of the possibility of developing fluorosis, which results in discolored and brown mottled teeth [84].

Fluoride has been shown to have caries preventive effects mainly through local topical application (post-eruptive effect); however, systemic fluoride administration may have pre-eruptive effects as well as cause dental fluorosis if fluoride is applied excessively during the tooth-development stage [85]. A silver fluoride and ammonia solution approved by the FDA is used to treat dental cavities. This remedy can be applied to the decayed lesion to stop decay or on the tooth's cavity-free area to avoid decay formation. It was discovered that Silver Diamine Fluoride (SDF) could stop primary tooth decay, manage hypersensitivity in the teeth, stop pit and fissure decay in newly erupting permanent teeth, disinfect contaminated root canals, and stop root caries in elderly people [86]. One disadvantage of this solution is that after using SDF, carious lesions become stained and turn black, which may not satisfy patients [87]. A biomimetic regenerative mixture based on HAp and calcium phosphate ion clusters, as the building blocks of amorphous calcium phosphate (ACP), has recently been developed. When applied to a carious lesion, it can induce epitaxial crystal growth through crystalline-amorphous mineralization. This solution, which is created by combining trimethylamine with calcium and phosphate minerals in an ethanol solution, has the ability to repair enamel up to 2.7 µm in thickness and has a structure that is exactly like that of native enamel [88]. Trimethylamine is a small, unstable organic molecule that may be removed, unlike polymeric stabilizers, which are permanent and can therefore affect the mechanical characteristics and integrity of the mineralized enamel [88].

#### Casein phosphopeptide (CPP-ACP nanocomplexes): a protein technology

Fluoride therapy can be highly effective on the smooth surface lesions; however, when treating pit and fissure caries and demineralized surfaces, it is less successful [84]. Mineralization can be enhanced by adding calcium and phosphate minerals. However, fluoride ions cannot be mixed with calcium phosphate ions in dental products because they tend to react with each other and form soluble compounds such as calcium fluoride (CaF_2_), which leads to a loss of bioavailability of fluoride ions [89]. One way to overcome this drawback is to use a dual-compartment toothpaste that has sodium fluoride (NaF) in one compartment and dicalcium phosphate dihydrate in the other. When the two toothpastes are combined before treatment, the fluoride delivery increases and the anticaries efficacy rises above that of dentifrice with just NaF [90].

Two technologies for calcium phosphate were also developed to improve the remineralization of enamel subsurface lesions and provide bioavailable and stable calcium, phosphate, and fluoride ions. These technologies are casein phosphopeptide stabilized amorphous calcium phosphate (CPP-ACP) and functionalized tricalcium phosphate (fTCP) [91]. Functionalized TCP is a calcium phosphate low-dose system that is added to a topical fluoride formulation in a single phase, either aqueous or non-aqueous. It acts as a barrier preventing TCP from prematurely interacting with fluoride and makes it easier to apply TCP to teeth in a targeted delivery [2]. Casein-phosphopeptide-stabilized amorphous calcium phosphate complexes (CPP-ACP) is a bioactive component of casein, has the ability to control supersaturated calcium and phosphorus ions in the oral environment [92]. As CPP was made from milk protein casein and has an astounding ability to both stabilize calcium phosphate in solution and to significantly raise the amount of calcium phosphate in dental plaque. By buffering the free calcium and phosphate ion activities, CPP-ACP helps to keep dental enamel supersaturated, decreasing demineralization while facilitating remineralization. Then the free calcium and phosphate ions travel through the enamel rods and re-form onto the apatite crystals [83].

Its most extensively researched remineralization technology was created by Eric Reynolds and associates at the University of Melbourne. It has since been used in tooth crèmes (GC Tooth Mousse™,GC Australasia and MI Paste™, GC America, Inc, Alsip, IL) and chewing gums (Recaldent gum™, GC Australasia and Trident White™,United States) [76]. A product with incorporated fluoride up to 900 ppm (GC Tooth Mousse Plus™, MI Paste Plus™). Using protein nanotechnology, amorphous calcium phosphate (ACP) nanoparticles are formed by combining certain phosphoproteins from bovine milk. The exact ratio is 96 phosphate ions to 144 calcium ions and six CPP peptides [76]. It is the product that is most popular and commonly used in clinical practice for white spot lesion [93]. It can be concluded that, in the treatment of early carious lesions, calcium-phosphate-based remineralization technology has shown promise as an adjuvant to fluoride therapy [94]. However, the fluoride and calcium phosphate nanocrystal protection for the enamel is restricted to the outside ∼30 µm of the tooth. Additionally, the produced HAp's mechanical and structural qualities are inferior to those of the original enamel [95].

#### Alpha tricalcium phosphate (TCP) and beta TCP (β-TCP)

Tricalcium phosphate exists in two forms: alpha and beta, and its chemical formula is Ca_3_(PO_4_)_2_. When human enamel is treated to high temperatures, alpha TCP appears. In aqueous conditions, it is a relatively insoluble substance (2 mg/100 mL in water) [96]. Calcium carbonate and calcium hydrogen phosphate can be combined to create crystalline beta TCP, which is a flaky, stiff powder that is produced by heating the mixture to over 1000 degrees Celsius for one day. The TCP particles can then be milled to change their average size. It is a component of Cerasorb® (CURASAN Co Ltd., Frankfurt, Germany), Bio-Resorb® (Oraltronics, Ilmenau, Germany), and Biovision® products (GmbH, Germany) [97]. TCP has been proposed as the most effective calcium ion release agent, even in a neutral pH environment, and it promotes the remineralization of the tooth surface [76, 98].

#### Nanoparticles for remineralization

Compared to microparticles, nanoparticles have superior ion release characteristics [83]. Nanomaterials are frequently incorporated to restorative materials as inorganic fillers, such as resin composites, to release calcium, phosphate, and fluoride ions for the remineralization of dental hard tissues, as it is challenging to use nanomaterials directly to remineralize teeth in the oral environment [77].

##### Nano-hydroxyapatite

Nanomaterials are one of the innovative materials that can restore the mineral content of tooth enamel. Particularly, nano-hydroxyapatite offers unique characteristics like higher surface energy, better biocompatibility, increased solubility, high surface area to volume ratio, and its ultrafine structure resembles biological apatite, and thus it can be used as a biomimetically alternative for enamel's natural mineral constituent [77]. The remineralization effect of nanohydroxyapatite is based on three mechanisms: creating a protective layer of “liquid enamel” on the teeth, restoring damage to the enamel with tiny nanohydroxyapatite crystals with a particle size of 20–80 nm, and accelerating the remineralization of hard tooth structures through the delivered phosphate and calcium ions [99]. According to Li and colleagues [2], n-HAP particles measuring 20 nm in size are well suited to the dimensions of the nanodefects on the enamel surface resulting from acidic erosion. These nanoparticles can firmly adhere to the demineralized enamel surface and prevent additional acid attack. The use of nano hydroxyapatite (nHAp) in conjunction with biopolymer scaffolds may potentially offer an additional viable method for tooth enamel remineralization and regeneration. Although the experimental results show promise, the n-HAP's mechanical and stability qualities are insufficient, and its clinical applicability is restricted due to its long mineral formation process (which can take from hours to days) [77].

##### ACP nanoparticles

These are tiny, spheroidal particles that range in size from 40 to 100 nm. ACP nanoparticles, as a supplier of calcium and phosphate ions, have been incorporated into glass ionomer cements, composite resins, and adhesives [83]. A study utilizing in situ caries models of humans showed that nanoACP-containing nanocomposites reduced demineralization at the restoration-enamel borders and resulted in less enamel mineral loss than the control composite [83]. According to Xu Zhang's [100] *in vitro* investigations, the remineralizing rate of Pchitosan-ACP complexes treatments was much higher than that of fluoride therapy.

##### Calcium fluoride nanoparticles

Considering the CaF_2_ nanoparticle (nano-CaF_2_) has a 20-fold greater surface area than standard glass ionomer cements, Xu HHK et al. [101] have demonstrated that the inclusion of nanoCaF_2_ increases the cumulative fluoride release compared to the fluoride release in traditional glass ionomer cements [77].

#### Gaseous ozone therapy

Using antibacterial agents is an additional strategy to protect the lesion from microorganisms and enhance remineralization by lowering the number of bacterial species linked to caries. Ozone is used to achieve this [102]. It has been demonstrated that ozone is efficient against gram-positive and gram-negative bacteria, viruses, and fungi with high remineralizing capabilities [103]. It is utilized to treat primary root caries, occlusal caries, and dentinal hypersensitivity because it is a potent oxidant and extremely bactericidal [104]. Additionally, ozone has the ability to remineralize dentin lesions. Research has been conducted both *in vivo* and *in vitro* on the effective application of ozone for caries treatment, cavity disinfection, lowering the quantity of caries-associated microorganisms in dental plaque, and caries lesion remineralization [78]. The effectiveness of three enamel remineralization techniques on first approximal caries was evaluated by Grocholewicz et al. [78]: a nano-hydroxyapatite gel, gaseous ozone therapy, and combination of a nano-hydroxyapatite gel and ozone, concluded that ozone therapy and nano-hydroxyapatite gel had some ability to remineralize the subsurface lesions of dentine and approximate enamel in premolar and molar teeth. Furthermore, when both techniques are used together, the results are superior to when either nano-hydroxyapatite or ozone therapy is used alone.

#### Sodium calcium phosphosilicate: bioactive glass

According to classifications, a bioactive material is one that causes the body to react favorably, especially when it bonds to host bone tissue and forms a calcium phosphate layer on a material surface [105]. As certain bioactive substances can cause the formation of calcium phosphate, they have been researched for potential uses in tooth remineralization [2]. A class of bioactive substance made up of calcium, sodium, phosphate, and silicate is called bioglass (BG). When they come into contact with bodily fluids, they become reactive and cause the particles' surface to become coated in calcium phosphate [105]. Studies conducted both *in vivo* and *in vitro* have demonstrated that BG particles as NovaMin™ (GSK Sensodyne; Brentford, Middlesex, United Kingdom) can be deposited onto dentine surfaces and then, by causing the development of carbonated HAP-like materials, occlude the dentinal tubules [79]. Nevertheless, the potential of bioglass for dental enamel de- and re-mineralization applications has not been extensively studied. Burwell et al. [106] looked into how BG affected the *in vitro* healing of enamel white-spot lesions. According to the results, after 10 days, BG plus 5000 ppm fluoride significantly increased remineralization compared to a treatment with 5000 ppm fluoride alone. However, scanning electron microscopy techniques continued to clearly reveal that demineralization resulted in the loss of prismatic enamel structure. In addition, when compared to a non-treatment control group in an *in vitro* study using human enamel, toothpaste containing 7.5% w/w BG did not significantly protect against the surface-softening effects of orange juice challenges [107].

#### Inorganic pastes

Dental pastes with inorganic ingredients like HAp can be used to create nanocrystalline structures to repair early caries lesions and form synthetic enamel without the requirement for prior excavation [108]. The HAp nanocrystals in the enamel are dissolved in the first step of the inorganic paste repair process. Then, in the middle step, the enamel apatite crystals quickly begin to grow again, and new HAp crystals nucleate due to the presence of ionic species in the mother solution's extremely acidic pH (pH < 2). In the final step, a layer of newly produced HAp crystals with identical crystallographic orientations and high crystallinity is formed in the interface, While the crystals in the dense paste are randomly orientated and low crystalline due to the high supersaturation. Thus, the newly synthesized HAp retains its mechanical and physiochemical properties in the about 20 μm thick contact also; no prism forms were seen [109]. Despite this, when applied to simulate carious lesions, toothpastes containing nano-HAp showed comparatively stronger remineralization capacity than TCP and fluoride toothpastes. They could also reduce the depth of the lesion and further produce a new apatite layer [110].

#### Enamel matrix proteins

Amelogenin, enamelin, ameloblastin, and amelotin are the primary enamel matrix proteins (EMPs) expressed by ameloblasts; of these, amelogenin is the most crucial for the mineralization of enamel [111]. Recombinant amelogenin has been used to study the mechanism underlying amelogenin's role in mineralization. The crystallization of calcium and phosphate, as well as the size, orientation, and elongation of apatite crystals inside prism formations, are all regulated by amelogenin and its self-assembly [111]. Research has shown that, in a dose-dependent way, the recombinant full length amelogenin (rP172) can promote the creation of ordered needle-like fluoridated HAp crystals under physiological conditions (Fig. 5b). For the density of crystal packing and remineralization, fluoride addition to amelogenin is also crucial [112]. Recombinant amelogenin can be used to remineralize the enamel in situ for early caries lesions; however, the clinical use of this approach is limited because amelogenin expression and purification are difficult, expensive, and time-consuming processes [112, 113].

Furthermore, it was discovered that the full-sequence amelogenin-remineralized layer had a poor affinity for the native enamel's surface [113]. Consequently, the biomimetic approach has made an effort to replicate the amelogenin-containing enamel matrix in a simple and cost-effective way, without using amelogenin's full length [113]. Without employing the entire length of amelogenin, a protein nanofilm known as the phase-transited lysozyme film that mimics the N-terminal amlogenin and is immobilized with a synthetic peptide based on the C-terminal amelogenin can cause the epitaxial formation of enamel crystals both *in vitro* and *in vivo*. This method has the potential to remineralize enamel with a highly organized structure and mechanical properties like those of native enamel. Therefore, it holds potential for use in clinical settings where patients rinse their mouths with a buffer that mimics N- and C-terminal amelogenin components daily to promote the synthesis of minerals and treat dental caries [113]. Biomimetic enamel matrix proteins (EMPs) made of modified leucine-rich amelogenin peptide and non-amelogenin analogue have demonstrated the ability to cause the regeneration of enamel crystals with prism- and interprism-like structures when applied to the surface of etched enamel [114].

#### Self-assembling peptides

The incorporation of self-assembling peptides in enamel regeneration is beneficial because they form fibrous structures with a high aspect ratio (diameter and length in nanometre and micrometre size ranges) and have clearly defined surface functional groups that aid in the nucleation of minerals by periodically repeating in the structure [115]. Furthermore, the 3D environment created by fibrils that is dynamically stable can regulate the formation and deposition of crystals [115]. These peptides can also be injected as fluids, which triggers in situ gelation and enables these structures for filling cavities with asymmetrical shapes [7]. The strong binding capacity of the self-assembling peptide named P11-4 (Ace-Gln-Gln-Arg-Phe-Glu-Trp-Glu-Phe-Glu-Gln-Gin-NH2) for calcium ions can aid in the nucleation and formation of needle-shaped HAp crystals, thus aiding in the remineralization of enamel. However, this peptide's efficacy is restricted to the early stages of dental caries, and the proteolytic destruction of self-assembling peptides may limit the clinical applications of these peptides and reduce their effectiveness on the tooth surface [116].

#### Dendrimers

Highly branching spherical polymers known as dendrimers have a well-defined structure made up of a central core surrounded by repeating branches [117]. Since these polymers have limited size distributions and dimensionless length scaling, they have been used as biomimetic artificial proteins in biomedical applications [117]. They can replicate the role of the organic matrix in the mineralization of enamel by acting as mimics of amelogenin [118]. The first synthetic dendrimer utilized for enamel mineralization by HAp crystallization was Poly (amido amine) (PAMAM). Functional groups, generational changes, and dendrimer concentration can regulate the HAp's size and form [119]. However, PAMAM dendrimers need to have a greater adsorption affinity to the enamel surface in order to promote in situ regeneration or mineralization [120]. In order to improve the binding capacity, PAMAM-COOH dendrimer was coupled to alendronate (ALN), a HAp-anchored agent that was demonstrated to be able to adsorb on the enamel's surface and recreate HAp crystals that resembled nanorods with uniform sizes, shapes, and perpendicular orientations on the enamel's acid-etched surface [120]. However, because of its complicated synthesis method, the commercialization and clinical applications of ALN-PAMAM-COOH are restricted. Furthermore, the combination of remineralization, antibacterial, and antibiofilm actions may be produced by loading the plant-based anticaries agent honokiol in PAMAM-COOH [121].

#### Hydrogels

Hydrogels are three-dimensional arrangements of hydrophilic polymers that have the ability to absorb large amounts of aqueous solutions and encapsulate biological agents [122]. The three most common hydrogels used in enamel tissue remineralization are gelatin, agarose gel, and chitosan [7]. Gelatin hydrogel was utilized to imitate enamel tissue formation due to the structural and chemical similarities between the enamel matrix and gelatin. To delay the precipitation of calcium phosphates, the gelatin gel can be supplied with buffered calcium and phosphate/fluoride solutions using a double-diffusion chamber [123]. It only takes a few weeks to create spherical fluorapatite-gelatine aggregates with needle-shaped subunits and chemical and morphological characteristics similar to those of natural enamel [124]. Considering gelatin is a thermosensitive substance that is in a solution state at 37 °C, which is the physiological temperature, the reaction should be carried out at 25 °C in order to keep the gelatin in the gel state [123]. In order to raise melting temperature of gelatin up to 40 °C, glycine was added; however, this can result in biosafety concerns if the gel remains in the buccal cavity for an extended period of time [125]. Using agarose gel, which has a sol–gel transition temperature of about 60 °C, was applied to solve the gelatin challenge. The findings demonstrated that the prism-like structures containing hexagonal HAp crystals were formed in the newly generated tissue, which has mechanical properties similar to enamel [125]. Chitosan was added to agarose gel to act as an enamel matrix imitating component and to control prism formation and reduced carbonation [126]. Moreover, chitosan has the ability to adhere to negatively charged surfaces like tooth enamel because of the positive charges on its surface. Thus, chitosan has the ability to penetrate into enamel structure, so, it can be used to deliver essential mineral content to deeper layers of carious lesion or prevent it from growing leading to significant advancements [127]. Also, chitosan has the ability to stabilize mineral ions and improve the creation of ordered crystals [127]. Nevertheless, hydrogels were used as a more straightforward model for enamel remineralization.

Silver-doped bioactive glass [128], and antimicrobial GKIIKLKASLKLL-NH2 (GL13K) peptides [129] are examples of bioactive and antimicrobial agents that can be added to hydrogels to create antimicrobial hydrogels that promote dental tissue regeneration or mineralization while preventing bacterial infections or treating cariogenic bacteria. In addition, hydrogels can be enhanced with organic components, such as proteinases and enamel proteins, to more closely mimic the organic matrix [125]. The more viable hydrogel-based approach can be achieved by incorporation of amelogenin proteins in the hydrogel in order to control the crystallization, the growth, and the orientation of HAp crystals [8]. Amelogenin-containing enamel repair systems were created using the chitosan hydrogel, which has unique adhesive and antibacterial properties [130]. However, further investigation is still required to improve these systems for clinical application.

#### Electrospun mats

Amorphous calcium phosphate/poly(vinylpyrrolidone) (ACP/PVP) fibrous membranes produced by the electrospinning technique were able to protect the tooth and act as a metastable calcium and phosphate ion reservoir [131]. The electrospun mats become hydrated and produce a mineralizing hydrogel when exposed to artificial saliva containing fluoride ions. This hydrogel can then be employed to facilitate and improve the remineralization of enamel by facilitating the crystallization of fluoridated hydroxyapatite, which has a thickness of 500 nm [131]. Furthermore, the ACP/PVP electrospun mat allowed for the plugging of the exposed dentin tubules. In regard to this, using this method may decrease dentin hypersensitivity [131].

## Future directions

In the realm of cell-based strategies for enamel regeneration, significant challenges remain in achieving native-like characteristics, structure, and control over the size and shape of regenerated enamel. A deeper understanding of the interaction between resident stem cells, biomolecular signals, and gene expression pathways involved in natural tooth development is essential for advancing enamel regeneration. Although much progress has been made, many aspects of epithelial-mesenchymal interactions and cell-scaffold mechanisms are still unclear. Key to this process is replicating mesenchymal-epithelial signaling through the co-culturing of dental epithelial stem cells and dental mesenchymal stem cells within dual-compartment 3D-printed scaffolds. This approach facilitates molecular signaling between the two cell types, promoting differentiation into odontoblasts and ameloblasts, ultimately leading to the co-regeneration of enamel and dentin.

Despite advancements, current materials and scaffold designs for enamel regeneration remain limited. To improve the creation of enamel with the desired structural and functional properties, there is a need for scaffolds incorporating more biomimetic and bioactive agents, as well as optimizing structural features such as pore diameters, interconnectivity, and degradation rates. Additionally, several challenges persist, including enhancing the homing of resident stem cells, prolonging the bioavailability of signaling molecules and bioactive compounds, controlling their release profiles, and ensuring a sterile environment. Further, developing scaffolds that support vasculature and can be applied to small defects, while effectively integrating regenerated enamel with remaining tooth structures, remains a crucial focus. Achieving these goals will require interdisciplinary collaboration between biologists, material scientists, and clinicians. In parallel, acellular approaches, such as enamel remineralization, may provide a more feasible solution for repairing lost or damaged enamel. One of the main challenges in remineralization is replicating the highly organized structural hierarchy of enamel, including its prism and interprismatic regions, as well as its mechanical properties. Advancements in biomimetic mineralization techniques may be realized by integrating nanotechnology with protein-based technologies, such as casein phosphopeptides and amelogenin-like proteins. Nanotechnology offers the potential to develop bioactive nanoparticles that enhance enamel remineralization by interacting with the enamel surface, guiding the deposition of minerals and proteins that mimic amelogenin, and supporting the formation of enamel-like structures.

## Conclusions

Enamel regeneration and mineralization represent a rapidly advancing frontier in dental research and regenerative medicine. Unlike other tissues, enamel, the outermost layer of teeth, lacks the ability to naturally regenerate once damaged due to the irreversible loss of ameloblasts after tooth formation. Despite this, recent advancements in materials science, stem cell research, and biotechnology have opened new avenues for enamel repair and remineralization. Both cell-based and cell-free strategies have been explored, each offering promising solutions to restore enamel. Nanotechnology, in particular, holds significant potential in the development of more effective and bioactive treatments for enamel restoration. Although further research is needed to develop therapies that faithfully replicate the biological, structural, and functional properties of native enamel, the field of regenerative dentistry is progressing rapidly. The integration of advanced biomaterials and innovative therapeutic techniques is bringing us closer to the realization of enamel regeneration as a routine clinical practice.

## Data Availability

The data supporting this review are from previously reported studies and datasets, which have been cited.
